# Discrepancy between effects of carbapenems and flomoxef in treating nosocomial hemodialysis access-related bacteremia secondary to extended spectrum beta-lactamase producing *klebsiella pneumoniae* in patients on maintenance hemodialysis

**DOI:** 10.1186/1471-2334-12-206

**Published:** 2012-09-05

**Authors:** Chih-Chao Yang, Shau-Hsuan Li, Feng-Rong Chuang, Chih-Hung Chen, Chih-Hsiung Lee, Jin-Bor Chen, Chien-Hsing Wu, Chien-Te Lee

**Affiliations:** 1 Division of Nephrology, Department of Internal Medicine, Kaohsiung Chang Gung Memorial Hospital and Chang Gung University College of Medicine, 123 Ta Pei Road, Kaohsiung city, Niao Sung District, 833, Taiwan; 2 Division of Hematology-Oncology, Department of Internal Medicine, Kaohsiung Chang Gung Memorial Hospital and Chang Gung University College of Medicine, Kaohsiung, Taiwan; 3 Division of General Medicine, Department of Internal Medicine, Kaohsiung Chang Gung Memorial Hospital and Chang Gung University College of Medicine, Kaohsiung, Taiwan

**Keywords:** Bacteremia, Carbapenems, Extended spectrum beta-lactamase, Flomoxef, Hemodialysis access, *Klebsiella pneumoniae*

## Abstract

**Background:**

Hemodialysis (HD) patients are susceptible to extended spectrum beta-lactamase (ESBL)-producing bacterial infections. Because the optimal treatment and clinical significance of ESBL-producing *Klebsiella pneumoniae* (ESBL-Kp) HD access-related bacteremia remain unclear, we conducted this retrospective study to determine the clinical outcomes of patients treated with either flomoxef or a carbapenem.

**Methods:**

The eligibility criterion was fistula or graft- or catheter- related ESBL-Kp bacteremia in patients on maintenance HD. The clinical characteristics and antibiotic management were analyzed. Outcome was determined by mortality resulting from bacteremia during the 14‐day period after the first positive blood culture for flomoxef-susceptible ESBL-Kp.

**Results:**

The 57 patients studied were predominantly elderly, malnourished, with a history of severe illnesses and broad-spectrum antibiotic use before the onset of bacteremia, and with severe septicemia as determined by the Pitt bacteremia score (PBS). The study population comprised 7 fistula, 8 graft, and 42 HD catheter-related bacteremia (CRB) cases, and the mortality rate was high (36/57, 63.2%) in these 57 patients. Of 42 patients with CRB, those in the deceased group (27/42, 64.3%) had significantly lower levels of serum albumin, longer prior hospital stay and duration of catheter-dependent HD, and higher PBS than patients in the survived group. Failure to receive effective antibiotics (flomoxef or a carbapenem) within 5 days after onset of bacteremia and treatment with flomoxef both significantly contributed to higher mortality. Multivariate analyses revealed that flomoxef use, PBS, and catheter-dependent HD >30 days were independently associated with increased mortality (OR, 3.52; 95% CI, 1.19–58.17, OR, 2.92; 95% CI, 1.36–6.26 and OR, 5.73; 95% CI, 1.21–63.2, respectively).

**Conclusions:**

Considering the high mortality rate, ESBL-Kp should be recognized as a possible pathogen in patients on maintenance HD at high risk of acquiring HD access infections associated with ESBL-producing bacteria. Carbapenems rather than flomoxef should be the therapy of choice in these critically vulnerable patients.

## Background

Hemodialysis (HD) access-related infections are a major cause of morbidity and mortality in HD patients; the responsible microorganisms, prevention and treatment strategies, and outcome have been assessed in earlier studies [1,2]. Gram-negative bacteria (GNB) have been reported in up to 33% of cases with catheter-related bacteremia (CRB), indicating that empiric antibiotic therapy should target both gram-positive and gram-negative organisms [2]. Ignoring the possibility of GNB, especially those producing extended spectrum beta-lactamase (ESBL), as mediators of HD access-related infections places a considerable number of patients at unwarranted risk.

Risk factors for the acquisition of ESBL infections have been well studied and include—but are not limited to—HD, old age, prolonged hospitalization, severe illness, poor nutritional status, treatment in an intensive care unit (ICU), and previous exposure to broad-spectrum antibiotics, with overuse of extended-spectrum cephalosporins in the hospital setting as one of the most important factors [3,4]. In addition to the risk of immunocompromise, HD patients may be rendered susceptible to ESBL-related infection by frequent vascular catheterization, catheter manipulation, and the need for broad-spectrum antibiotics during prolonged hospitalization, especially in the ICU setting.

The currently recommended therapy for infection caused by ESBL-producing organisms consists of carbapenems [4,5]. Cephamycins (i.e., cefmetazole, cefotetan, and flomoxef), characterized by their 7-α-methyoxy β-lactam, have been reported to be highly active in vitro against both low inocula (10^5^–10^6^ cfu/mL) and high inocula (10^7^–10^8^ cfu/mL) of TEM- or SHV-producing Enterobacteriaceae [6]. Unfortunately, the use of cephamycins is not universal but limited in some countries. In addition, many controversies about optimal treatment exist, and few clinical reports comparing the treatment efficacy of cephamycins and carbapenems have been published [7].

The vast majority of *Klebsiella pneumoniae (K. pneumoniae)* infections are associated with hospitalization, and ESBL-producing bacteria are reported to comprise a significant percentage of nosocomial *K. pneumoniae* strains [8]. In a retrospective study, treatment with either flomoxef or a carbapenem in patients with flomoxef-susceptible ESBL-producing *K. pneumoniae* (ESBL-Kp) bacteremia was reported to be similarly effective [9]. However, their clinical effectiveness and most appropriate antibiotic prescription strategies for HD access-related ESBL-Kp bacteremia in patients on maintenance HD (MHD) have not yet been established. We conducted this 7-year retrospective study to compare the clinical outcomes of patients treated with either flomoxef or a carbapenem, and to gain a better understanding of the clinical significance and impact of HD access-related ESBL-Kp bacteremia in patients on MHD.

## Methods

### Study population and design

This retrospective study was conducted in a tertiary care hospital with 950 MHD patients treated in the outpatient department*.* MHD patients admitted during the 7-year period from January 2001 to December 2007 who developed a nosocomial HD access-related infection secondary to ESBL-Kp were included. The eligibility criterion was HD access, including arteriovenous fistula or graft- or catheter-related ESBL-Kp bacteremia. Only adult patients with flomoxef-susceptible ESBL-Kp bacteremia who were treated with either flomoxef or a carbapenem (meropenem or imipenem) were included. For each included patient, the prescribed flomoxef or a carbapenem was administered for at least 2 days, starting within 5 days after receiving finalized blood culture results. The use of either flomoxef or a carbapenem was left to the discretion of the attending physician in each case. Details of the patients’ clinical course, biochemical data, sites of ESBL-Kp infection (blood, HD catheter tip, pus from HD catheter exit site, and pus from fistula or graft wounds), and outcome were obtained from medical charts. Individual patient without blood culture that grew ESBL-Kp was excluded because, in the absence of concurrent blood cultures from the peripheral vein, there is a risk of enrolling patients simply representing colonization. Severely ill patients who died rapidly without receiving a carbapenem or flomoxef for at least two days were also excluded. Variables used for the assessment of severity of illness included Pitt bacteremia score (PBS), ICU stay at the time of bacteremia, and length of prior hospital stay (LOS). Mortality resulting from bacteremia within 14 days after the first positive blood culture that grew flomoxef-susceptible ESBL-Kp was used to determine the outcome.

The study was approved by the institutional review board of Kaohsiung Chang Gung Memorial Hospital with the study ID: CGMH-IRB-100-2452B.

### Microbiology

All *K. pneumoniae* isolates were identified by standard methods, and the presence of ESBLs was evaluated using the Clinical and Laboratory Standards Institute criteria for ESBL screening and disc confirmation test [10]. Minimum inhibitory concentrations (MICs) ≤ 4 mg/L for meropenem and ≤ 8 mg/L for flomoxef were considered to indicate susceptibility [11,12].

### Definitions

ESBL-Kp bacteremia was defined by the isolation of ESBL-Kp from blood cultures. Nosocomial bacteremia was defined as bacteremia occurring >48 h after admission to the hospital. HD access infections were defined as local signs (pus or redness) at the vascular access site with or without a positive culture of catheter tip, pus, or a positive blood culture with no known source other than the vascular access. Empiric antibiotic was defined as antibiotic therapy started at the time blood cultures were drawn. Effective antibiotics for flomoxef-susceptible ESBL-Kp bacteremia included carbapenems and flomoxef. Significant underlying disease was defined as a medical history of diabetes mellitus, liver cirrhosis, malignancy, and congestive heart failure.

### Statistical analyses

All statistical analyses were performed using the Statistical Package for Social Science program (SPSS for Windows, Version 11.5; SPSS, Chicago, IL). Continuous variables were each expressed as the mean ± SD and were analyzed using the Student *t* test. The statistical difference in the frequency of occurrence of the variants in patients with HD catheter-related bacteremia between survived and deceased subgroups was assessed by the chi-square test or Fisher’s exact test. A logistic regression model was used to estimate the effects of multiple factors associated with mortality in the univariate analyses. Variables with *P* ≤ 0.1 in univariate analysis between patients of deceased and survived groups were entered for further assessment. Estimated ORs and 95% CI were obtained from this model. For all analyses, two-sided tests of significance were used with P < 0.05 considered significant.

## Results

A total of 57 MHD patients with HD access-related bacteremia who met the criteria were identified during the study period. The clinical courses and characteristics of these patients are summarized in Table 1. In addition to 42 patients with HD CRB, there were 7 patients with fistula and 8 patients with graft-related bacteremia. Most of these patients were male and elderly, with a history of severe illness that included ICU stay, shock, or intubation during their prolonged hospitalization prior to the onset of bacteremia. Significant underlying diseases and prior use of broad-spectrum antibiotics were also highly prevalent. Most patients were initially hospitalized due to the onset of an infectious disease (34/57, 59.6%). At the onset of bacteremia, poor nutritional status and critical illness, defined as PBS greater or equal to 4 points, were noted in these patients. Twenty-nine (29/57, 50.9%) patients were accommodated in an ICU at the time of the detection of bacteremia. The overall mortality rate was high (36/57, 63.2%), and patients with graft infection had the highest PBS and the highest rate of mortality (6/8, 75%). With regard to the use of empiric antibiotics, the majority (36/57, 63.2%) did not receive effective antibiotic therapy, consisting of either flomoxef or a carbapenem within 5 days after the onset of bacteremia. A total of 50.9% (29/57) patients were treated with flomoxef and 49.1% (28/57) with carbapenems as effective antibiotics.

**Table 1:** Comparisons of demographic and clinical data between the groups of different HD access related ESBL-Kp bacteremia

**Source of infection**	**HD catheter**	**Fistula**	**Graft**	**ALL**
**Case No**	42	7	8	57
**Male (%)**	33 (78.6)	6 (85.7)	6 (75)	45 (78.9)
**Age, years**	64.8 ± 10.0	61 ± 10.5	65.1 ± 7.8	64.4 ± 9.5
**Patients aged >65 years (%)**	24 (57.1)	3 (42.9)	5 (62.5)	32 (56.1)
**Admission for infectious diseases (%)**	23 (54.8)	5 (71.4)	6 (75)	34 (59.6)
**Significant underlying diseases (%)**				
Diabetes mellitus	15 (35.7)	4 (57.1)	7 (87.5)	26 (45.6)
Liver cirrhosis	5 (11.9)	1 (14.3)	2 (25)	8 (14)
Congestive heart failure	10 (23.8)	3 (42.9)	5 (62.5)	18 (31.6)
Malignancy	4(9.5)	1 (14.3)	1 (12.5)	5 (8.8)
**Comorbid conditions (%)**				
Poor nutrition^**a**^	42 (100)	7 (100)	8 (100)	57 (100)
Prior antibiotic use^**b**^	37 (88.1)	6 (85.7)	7 (87.5)	50 (87.7)
Previous severe illness^**c**^	35 (83.3)	6 (85.7)	6 (75)	47 (82.5)
Prolonged (>30 days) hospitalization	33 (78.6)	5 (71.4)	6 (75)	44 (77.2)
Prior use of 3^rd^-generation cephalosporin	27 (64.3)	5 (71.4)	6 (75)	38 (66.7)
ICU stay at or after the onset of bacteremia	20 (47.6)	4 (57.1)	5 (62.5)	29 (50.9)
**Pitt bacteremia score**	5.29 ± 1.67	5 ± 1.73	6.38 ± 2.26	5.4 ± 1.78
**ABx after onset of bacteremia**^**d**^				
Effective ABx within 5 days (%)	15 (35.7)	3 (42.9)	3 (37.5)	21 (36.8)
Use flomoxef/IMP /MEP as effective ABx(%)	19(45)/13(31)/ 10(24)	4(57)/3(43)/ none	6 (75)/none/ 2(25)	29(51)/16(28)/ 12(21)
**Mortality (%)**	27 (64.3)	3 (42.9)	6 (75)	36 (63.2)

^a^ albumin < 3.5 g/dL; ^**b**^ including extended-spectrum cephalosporins, aztreonam, fluoroquinolones, trimethoprim/sulfamethoxazole, or aminoglycosides; ^**c**^ includes shock, intubation and ICU stay; ^**d**^ Abx: antibiotics, IMP: imipenem, MEP: meropenem.

In addition to antibiotic treatment, surgical interventions (wound debridement and/or removal of infected fistula or graft) were performed in 7 of 15 patients with fistula or graft infection. All HD catheters related to ESBL-Kp bacteremia in 42 patients were removed after the onset of bacteremia. The extent and perioperative risk of surgery may affect survival of patients with fistula or graft infection but their effects were difficult to estimate. We believe bias and confounding factors could be reduced if we only analyzed 42 patients, all with catheter-related bacteremia.

Most of the 42 patients with HD CRB were elderly, exhibited low levels of serum albumin and hemoglobin at the onset of bacteremia, and had a history of prolonged hospitalization. Patient demographics are summarized in Table 2. Of the 42 patients included in the study, those in the deceased group had significantly lower levels of serum albumin, a longer hospital stay, and catheter-dependent HD. The mortality rate was high (27/42, 64.3%). Concerning the severity of the septicemia, a significantly higher PBS and a trend toward a longer ICU stay were noted in the deceased group. Antibiotic strategies also significantly affected the outcome. Treatment with flomoxef or failure to receive effective antibiotic therapy within 5 days after the onset of bacteremia was associated with increased mortality.

**Table 2:** **Comparisons of demographic and clinical data between the deceased and survived groups of the 42 patients with CRB; Variables with a *p*-value < 0.1 by univariate analysis were subjected to multivariate analysis**

**Variable**	**Survived group**	**Deceased group**	***P***	**Total**
	**n = 15**	**n = 27**		**n = 42**
**Mean age, years**	63.2 ± 7.6	65.7 ± 10.3	NS	64.8 ± 10.0
**Male (%)**	12/15 (80)	21/27 (77.8)	NS	33/42 (78.6)
**Patients aged >65 years (%)**	8/15 (53.3)	16/27 (59.3)	NS	24/42 (57.1)
**Flomoxef treatment (%)**^**a**^	3 (20)	16 (59.3)	0.009	19 (45.2)
**Treatment within 5 days**	9 (60)	6 (22.2)	0.021	15 (35.7)
**Pitt bacteremia score, mean ± SD**^**a**^	4.27 ± 1.03	5.85 ± 1.7	0.002	5.29 ± 1.67
**Serum albumin(g/dL), mean ± SD**	2.70 ± 0.31	2.12 ± 0.42	0.046	2.33 ± 0.61
**Hemoglobin(g/dL), mean ± SD**	8.98 ± 0.71	9.13 ± 1.92	NS	9.08 ± 1.56
**Hospital days before onset, mean ± SD**	49 ± 35.8	116.9 ± 87.5	0.039	92.6 ± 79.6
**No. of hospital days before onset > 30 (%)**	8/15 (53.3)	23 (85.2)	0.034	30/42 (71.4)
**Duration of catheter-dependent HD(days)**	24.5 ± 9.2	43.6 ± 12.3	0.048	36.6 ± 10.2
**No. of catheter-dependent HD days > 30 (%)**^**a**^	4 (26.7)	18 (66.7)	0.023	22 (52.4)
**ICU stay at the time of bacteremia**	4 (26.7)	16 (59.3)	0.058	20 (47.6)

^a^ Variable with a *p*-value < 0.05 by multivariate analysis, CRB: catheter-related bacteremia.

Variables that were determined to be significantly associated with mortality at 14 days after the first positive blood culture result by univariate analysis were subjected to multivariate analysis. Flomoxef use, higher PBS, and catheter-dependent HD > 30 days were independently associated with increased mortality (OR, 3.52; 95% CI, 1.19–58.17; OR, 2.92; 95% CI, 1.36–6.26 and OR, 5.73; 95% CI, 1.21–63.2, respectively).

## Discussion

There is limited information regarding the clinical characteristics associated with HD access-related ESBL-Kp bacteremia in MHD patients. Most of the patients with bacteremia in the present study were elderly, malnourished, and had a history of severe illness and prolonged hospitalization. These patients were also critically ill, as determined by PBS, and had high prevalence rates of significant underlying diseases and ICU stay at the time of the onset of bacteremia; therefore, they had a high mortality rate(36/57, 63.2%). Although broad-spectrum antibiotic treatment prior to the onset of bacteremia was also highly prevalent, its contribution to the acquisition of ESBL infection and effect on patient outcome were not analyzed; this might contribute to an epidemiological bias. Only 36.8% of these critically ill patients received empiric effective antibiotics within 5 days after the first positive blood culture for ESBL-Kp, which may have contributed to the high mortality rate.

Previous studies have demonstrated vascular access accounts for 48–73% of bacteremia in HD patients [2]. In the present study, both longer LOS and a lower serum albumin level were associated with high mortality in patients with CRB. Prolonged hospitalization has been recognized as a risk factor for ESBL infection in prior studies, which reported a mean time to acquisition of ESBL-Kp bacteremia of more than 1 month [13]. Hypoalbuminemia was found to increase the likelihood of a recurrent episode of CRB among patients treated for an initial CRB [1]. In the present study, the duration of catheter-dependent HD before bacteremia was on an average longer than 1 month, and catheter-dependent HD ≥ 30 days was a significant independent risk factor for increased mortality by multivariate analysis. These findings suggest that shortening the duration of catheter-dependent HD may decrease the probability of CRB secondary to ESBL-producing bacteria and improve the prognosis. It is not surprising to find that higher PBS was independently associated with increased mortality. There was also a trend toward a longer ICU stay at the time of bacteremia in the deceased group. Because MHD patients with HD access-related ESBL-Kp bacteremia were mostly immunocompromised and critically ill, their mortality rate was determined by these parameters, which reflected the severity of the septicemia.

For ESBL infections, there are both a higher clinical failure rate [14] and mortality rate [13] associated with cephalosporin treatment. Furthermore, ESBL-producing bacteria are frequently resistant to many classes of beta-lactam and non-beta-lactam antibiotics, including cefepime [15], beta-lactam/beta-lactamase inhibitor combinations, fluoroquinolones [16], and aminoglycosides [17]. The emergence of multi-drug resistance in these virulent pathogens has significantly hampered the effort to devise effective empiric or directed antibiotic treatment regimens, and is associated with high treatment failure rates. Unfortunately, most of our patients initially received ineffective empiric antibiotics, including extended-spectrum cephalosporins, which increased the difficulty of further treatment and led to a high treatment failure and mortality rates.

Although carbapenems have been recommended for the treatment of infections caused by ESBL-producing organisms, extensive use of these antibiotics may result in the outbreak of infections related to carbapenem-resistant strains such as *Acinetobacter baumanii*[18], *Pseudomonas aeruginosa*[19], and *Stenotrophomonas maltophilia*[20], and it poses challenging infection-control issues. Flomoxef is unique among cephamycins in having a difluoromethylthio-acetamido group at position 7, which improves its in vitro activity against ESBL-producing Enterobacteriaceae [6,21]. Flomoxef was also shown to be as clinically effective as a carbapenem for the treatment of flomoxef-susceptible ESBL-Kp bacteremia in terms of clinical outcome and comparable results of time-kill studies regardless of the inoculum size of 10^5^ or 10^7^ cfu/mL [9]. Although this study was limited by its retrospective design and small sample size, it provides insight into the possibility of using a cephamycin for infections caused by ESBL-producing bacteria. However, the efficacy of these agents for the treatment of HD access-related ESBL-Kp bacteremia in MHD patients is still unclear.

The use of effective antibiotics such as carbapenems during the 5-day period after the onset of bacteremia due to an ESBL-producing organism has been reported to be associated with lower mortality [22]. In the present study, treatment with effective antibiotics within 5 days after the onset of bacteremia and the use of carbapenems improved the prognosis in patients with CRB. The choice of antibiotics is critical, in the light of our finding that treatment with flomoxef was an independent risk factor for increased mortality in these vulnerable patients by multivariate analyses. The lower percentage of patients receiving effective empiric antibiotics may reflect our collective unawareness of the significance of these emerging strains and the very limited information regarding this issue.

## Conclusion

This is the first study to report the clinical implications of nosocomial HD access-related ESBL-Kp bacteremia. We stress that ESBL-Kp should be considered as a possible pathogen in HD access-related infections. Because of the high antimicrobial resistance and high mortality rate in these more vulnerable and critically ill patients, we suggest carbapenems rather than flomoxef as the first choice for empiric or directed therapy in HD patients at high risk for HD access-related ESBL-Kp infections. Shortening the duration of catheter-dependent HD also improved the outcome.

## Competing interests

The authors declare that they have no competing interests.

## Authors’ contributions

CCY was responsible for the literature search, study design, data collection, data analysis, data interpretation, and writing the manuscript. SHL contributed to the literature search, data collection, statistical analysis and data interpretation. FRC contributed to the literature search and data collection. CHC contributed to the literature search and data collection. CHL contributed to the literature search and data collection. JBC contributed to the literature search and data collection. CHW was responsible for the literature search, study design, data collection, data analysis, data interpretation, and article revision. CTL was responsible for the literature search, study design, data collection, data analysis, data interpretation, and article revision. All authors read and approved the final manuscript.

## Pre-publication history

The pre-publication history for this paper can be accessed here:

http://www.biomedcentral.com/1471-2334/12/206/prepub
